# Comparison of two visual angiographic perfusion grades in acute myocardial infarction

**DOI:** 10.1080/03009730902990453

**Published:** 2009-09-07

**Authors:** Tamás Ungi, Viktor Sasi, Imre Ungi, Tamás Forster, András Palkó, Attila Nemes

**Affiliations:** ^1^Department of Radiology, Medical Faculty, Albert Szent-Györgyi Clinical Center, University of SzegedSzegedHungary; ^2^Division of Invasive Cardiology, 2nd Department of Medicine and Cardiology Center, Medical Faculty, Albert Szent-Györgyi Clinical Center, University of SzegedSzegedHungary

**Keywords:** Acute myocardial infarction, angioplasty, myocardial perfusion

## Abstract

**Introduction:**

Prognosis after opening the obstructed coronary artery in acute myocardial infarction (AMI) is influenced by several factors. In routine clinical practice, revascularization is considered to be successful when the restoration of epicardial blood-flow is complete. However, the patent epicardial artery does not always provide functional recovery in the myocardium. There are two visual angiographic grades to assess myocardial perfusion: myocardial blush grade (MBG) and TIMI myocardial perfusion grade (TMP). The aim of our study was to compare these two parameters, how they correlate with short-term indicators of myocardial damage.

**Patients and methods:**

The two visual grades were assessed along with enzymatic infarct size as creatine kinase release (CK), echocardiographic left ventricular ejection fraction (LVEF), and ST-segment resolution (STR) in 62 patients with acute myocardial infarction and successful revascularization.

**Results:**

Better correlation was found with TMP in case of all clinical parameters (CK: *R*= − 0.687, *P*<0.001; LVEF: *R*=0.586, *P*<0.001; STR: *R*=0.574, *P*<0.001). MBG also showed significant correlations with clinical measurements, except for enzymatic infarct size (CK: *R*=− 0.062, *P*=0.626; LVEF: *R*=0.389, *P*=0.002; STR: *R*=0.348, *P*=0.006).

**Conclusion:**

Our findings suggest that the clearance of the dye (described by TMP) is more characteristic to myocardial recovery after AMI, than maximal contrast density (described by MBG) in the clinical practice.

## Introduction

Functional recovery of the myocardium after acute myocardial infarction (AMI) and recanalization of the occluded coronary artery by primary coronary intervention (PCI) are influenced by several factors including pain-to-balloon time, myocardial capacity for regeneration, and microembolization distal to the thrombus. In routine clinical practice, reperfusion treatment is considered to be successful when restoration of the epicardial blood-flow is complete.

In current clinical practice, successful recanalization of AMI is described by an increase in blood-flow in the epicardial artery characterized by thrombolysis in myocardial infarction (TIMI) flow grade (1,2). However, myocardial reperfusion does not depend only on the epicardial flow described by TIMI flow grade in case of successful recanalization (TIMI 3 flow). In some cases, only the reduced myocardial perfusion indicates permanent damage of affected area after successful revascularization (3–5). The so-called ‘no-reflow’ phenomenon, an open epicardial artery without flow into the myocardium, predicts complications. Therefore, assessment of myocardial perfusion has great importance in risk stratification after AMI and successful PCI (6).

Van 't Hof et al. described the first angiographic grading method for the assessment of myocardial perfusion (7). A new parameter was introduced, the myocardial blush grade (MBG), based on the observation of maximal contrast density at the area at risk compared to healthy areas. Gibson et al. suggested another grading scale describing temporal characteristics of myocardial contrast signal called TIMI myocardial perfusion grade (TMP) (8). TMP classifies reperfusion based on the dynamics of contrast clearance.

After demonstrating a high correlation between recovery of myocardial function after AMI and visual perfusion grading (both MBG and TMP), they have been widely used as an end-point in clinical studies evaluating effectiveness of interventional tools and other therapeutic methods. However, no reports have been found to evaluate and compare these visual grading scales on the same population.

## Patients and methods

### Study population

The present prospective study comprised 62 patients with ST-segment elevation myocardial infarction (STEMI) treated by PCI. Patients were enrolled by the following inclusion criteria: 1) pain-to-balloon time <12 h; 2) ST-segment elevation >0.1 mV in at least three contiguous electrocardiographic leads; 3) occluded coronary artery on the diagnostic angiogram (TIMI grade 0 flow) that is at least 2 mm in diameter and supplies a medium to large myocardial area; 4) successfully opened coronary artery after PCI (TIMI grade 3 flow); 5) ability of the patient to co-operate. Patients who were unconscious, or showed signs of cardiogenic shock, or had visible collateral circulation to the infarct-related myocardial region have been excluded from the study. Demographic and clinical data of patients are shown in Table I. Informed consent was obtained from each patient, and the study protocol conformed to the ethical guide-lines of the 1975 Declaration of Helsinki, as reflected in a-priori approval by the human research committee of the University of Szeged.

**Table I.** Clinical features of the patients.

Sample size (*n*)	62
Male gender (%)	43 (70%)
Age (years±SD)	60±6
Diabetes (%)	6 (10%)
Hypertension (%)	45 (73%)
Smoking (%)	37 (60%)
Infarct-related artery:	
Left anterior descending coronary artery (%)	31 (50%)
Left circumflex coronary artery (%)	11 (17%)
Right coronary artery (%)	20 (33%)

### Technical features of coronary angiography

Angiographic parameters were assessed on an AI 1000 (GE Healthcare, Chalfont St. Giles, Buckinghamshire, United Kingdom) work station. For the determination of perfusion grades of the area at risk, the final angiogram of the coronary intervention was performed under the following criteria: 1) motion of the patient or the table should be avoided; 2) the patient should hold breath for the time of the recording; 3) the field of view is to be set to contain the whole supplied area of the vessel of interest. All coronary angiograms met with these criteria. The same non-ionic contrast material (Visipaque, 320 mg/mL iodine) was used for all angiograms injected by a manual injector, the contrast quantity was 6.84±0.97 mL (mean±SD), the injection rate was 3.04±0.34 mL/s. Angiograms were recorded on an Innova 2000™ (GE Healthcare) system, and images were stored in 512×512 size 8-bit, greyscale, uncompressed format.

### Echocardiographic, electrocardiographic, and enzymatic measurements

Echocardiographic measurements were performed 3 days after the primary PCI to assess left ventricular ejection fraction (LVEF). Twelve-lead electrocardiograms were recorded at the beginning of PCI and 90 minutes later. ST-resolution was defined as a decrease of ST-segment at 90 minutes compared to the first measurement in the lead with highest ST-segment elevation, expressed as percentage of initial ST-elevation. Blood creatine kinase (CK) enzyme levels were measured 6, 12, 24, and 48 hours after the PCI. These four measurements were summed up to assess total enzymatic infarct size.

### Analysis of myocardial perfusion

MBG and TMP were graded in a random sequence independently by two cardiologists experienced in analysis of angiograms, who were blinded to all other clinical data. Final values for each patient were based on consensus between the observers. Patient groups of grades 0 and 1 of both MBG and TMP were united in statistical analysis, due to identical definitions in MBG 0 and TMP 1. See Table II for definitions used for grading of angiograms.

**Table II.** Definitions of perfusion grades.

Grade	Myocardial blush grade	TIMI myocardial perfusion grade
0/1	No or minimal contrast density, or persistent density on subsequent image acquisition.	No or minimal contrast density, or persistent density on subsequent image acquisition.
2	Moderate contrast density, but less than that obtained during angiography of a contralateral or ipsilateral non-infarct-related coronary artery.	Contrast density is strongly persistent and diminishes minimally or not at all during three cardiac cycles of the wash-out phase.
3	Normal contrast density, comparable with that obtained during angiography of a contralateral or ipsilateral non-infarct-related coronary artery.	Contrast density is minimally persistent after three cardiac cycles of the wash-out phase.

TIMI = thrombolysis in myocardial infarction; MBG = Myocardial blush grade; TMP = TIMI myocardial perfusion grade.

### Statistical analysis

All statistical tests were performed with MedCalc software package (MedCalc Software, Mariakerke, Belgium). A value of *P <*0.05 was considered to be statistically significant. Our results were obtained by Spearman's rank correlation at a confidence interval of 95%.

## Results

**Figure 1.**
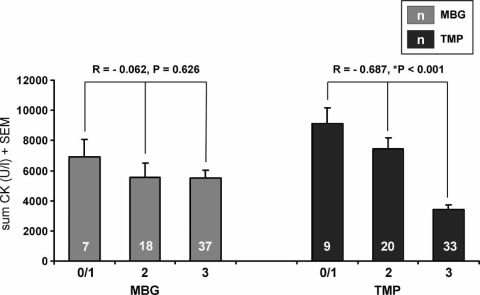
Rank correlation between summed serum creatine kinase (CK) levels and visual perfusion grades (MBG and TMP). *R*=Spearman correlation coefficient; *n*=sample size; MBG = myocardial blush grade; TMP = TIMI myocardial perfusion grade.

**Figure 2.**
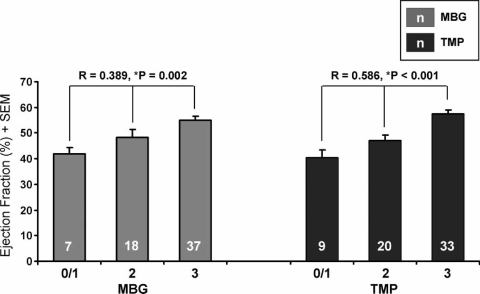
Rank correlation between ejection fraction measured 3 days after AMI and visual perfusion grades (MBG and TMP). *R*=Spearman correlation coefficient; *n*=sample size; AMI = acute myocardial infarction; MBG = myocardial blush grade; TMP = TIMI myocardial perfusion grade.

**Figure 3.**
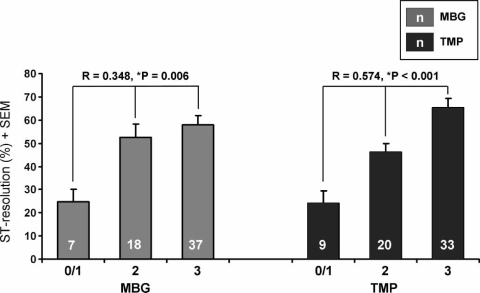
Rank correlation between ST-segment resolution measured 90 minutes after AMI and visual perfusion grades (MBG and TMP). *R*=Spearman correlation coefficient; *n*=sample size; AMI = acute myocardial infarction; MBG = myocardial blush grade; TMP = TIMI myocardial perfusion grade.

All patients underwent a successful recanalization of the occluded vessel and achieved <50% residual stenosis within 12 hours from the onset of symptoms. Enzymatic infarction size as expressed by sum of CK release had a significant negative correlation (*R*= − 0.687, *P*<0.001) with TMP, but not with MBG (*R*= − 0.062, *P*=0.63) as shown in Figure 1. A positive, significant correlation was found between echocardiographic LVEF measured 3 days after PCI and both MBG (*R*=0.389, *P*=0.002) and TMP (*R*=0.587, *P*<0.001). TMP showed a stronger correlation as shown in Figure 2. ST-segment resolution as percent decrease of initial ST-elevation also correlated with MBG (*R*=0.348, *P*=0.006) and had a stronger relation to TMP (*R*=0.574, *P*<0.001) (Figure 3), which is consistent with previous findings.

## Discussion

The main finding of the present study is that TMP has a stronger correlation to enzymatic infarction size, LVEF, and ST-resolution than MBG has; however, both grades show the same tendency when compared to other clinical parameters of AMI patients after PCI. These results suggest that clearance dynamics of dye from the microvasculature (classified by TMP grade) gives more precise information on indicators of myocardial viability than absolute density of the myocardial blush (classified by MBG).

In animal models, the change of density as a function of time was found to correlate better with effective tissue perfusion than the absolute value of contrast density. Pijls et al. investigated the relationship between epicardial blood-flow, measured with Doppler flowmetry, and densitometric parameters on the angiogram (9). They found that the most informative parameter of the time-density curve, registered in the area at risk, is mean transit time. Haude et al. have supported this result using coloured microspheres to directly measure myocardial perfusion (10). In these studies it was also found that a strong correlation between time to maximal contrast density and absolute blood perfusion exists, whereas maximal contrast density was poorly associated with real perfusion. These results show that even in standardized, experimental circumstances, analysis of the contrast density as a function of time is more informative and therefore more important than the assessment of maximal contrast density. These conclusions are consistent with the findings of the present study, that contrast clearance is more informative than contrast density.

The importance of this is further emphasized by the difference in back-flow of the contrast material to the aorta in different angiograms, which may influence maximal contrast density as observed in the myocardial area. Visual evaluation of maximal density may also be corrupted by automatic image processing of the angiographic computer system (done in order to better visualize epicardial arteries). Image processing results in different brightness of images recorded in different projections, which further limits comparison of affected and healthy areas.

In contrast with these findings, MBG is more widely used in the literature than TMP to evaluate new interventional devices (11–13) and other therapeutic methods (14). This may be explained by the historical importance of MBG, since it was the first method to evaluate myocardial tissue perfusion by videodensitometry. TMP was introduced only 2 years later. Since there was no study comparing the usefulness of the two perfusion grades, there was no evidence on the better prognostic value of TMP grade. Our results suggest that TMP should be used in clinical practice, rather than MBG. Computerized image processing may help improving the consistency of these gradings in the future, and thus assessing myocardial tissue perfusion may become wide-spread in routine clinical practice during acute PCI.

As a limitation of this study, it should be noted that the extent of necrosis in AMI is a function of several factors, e.g. pain-to-balloon time, existing collaterals, and technical success of the procedure. This should be taken into consideration when discussing the results of this single-centre study, with a limited number of patients. Our patient population was younger, with few diabetics, smokers, and hypertensives, compared to other studies. A follow-up study on these patients, as well as multi-centre studies, should be carried out to provide further evidence on our conclusions.

In conclusion, examination of contrast density in the myocardium with coronary angiography provides important information on myocardial viability and, with this, on the evaluation of recanalization success. Dynamics of contrast density is more informative than the level of maximal contrast density. Since visual estimation is subjective, angiographic evaluation by experienced and independent physicians is of primary importance.
